# Supplementary figures and images for: Diabetes induced decreases in PKA signaling in cardiomyocytes: The role of insulin
Source: PLoS One. 2020 Aug 20;15(8):e0231806. doi: 10.1371/journal.pone.0231806 (PMC7444578; doi:10.1371/journal.pone.0231806)

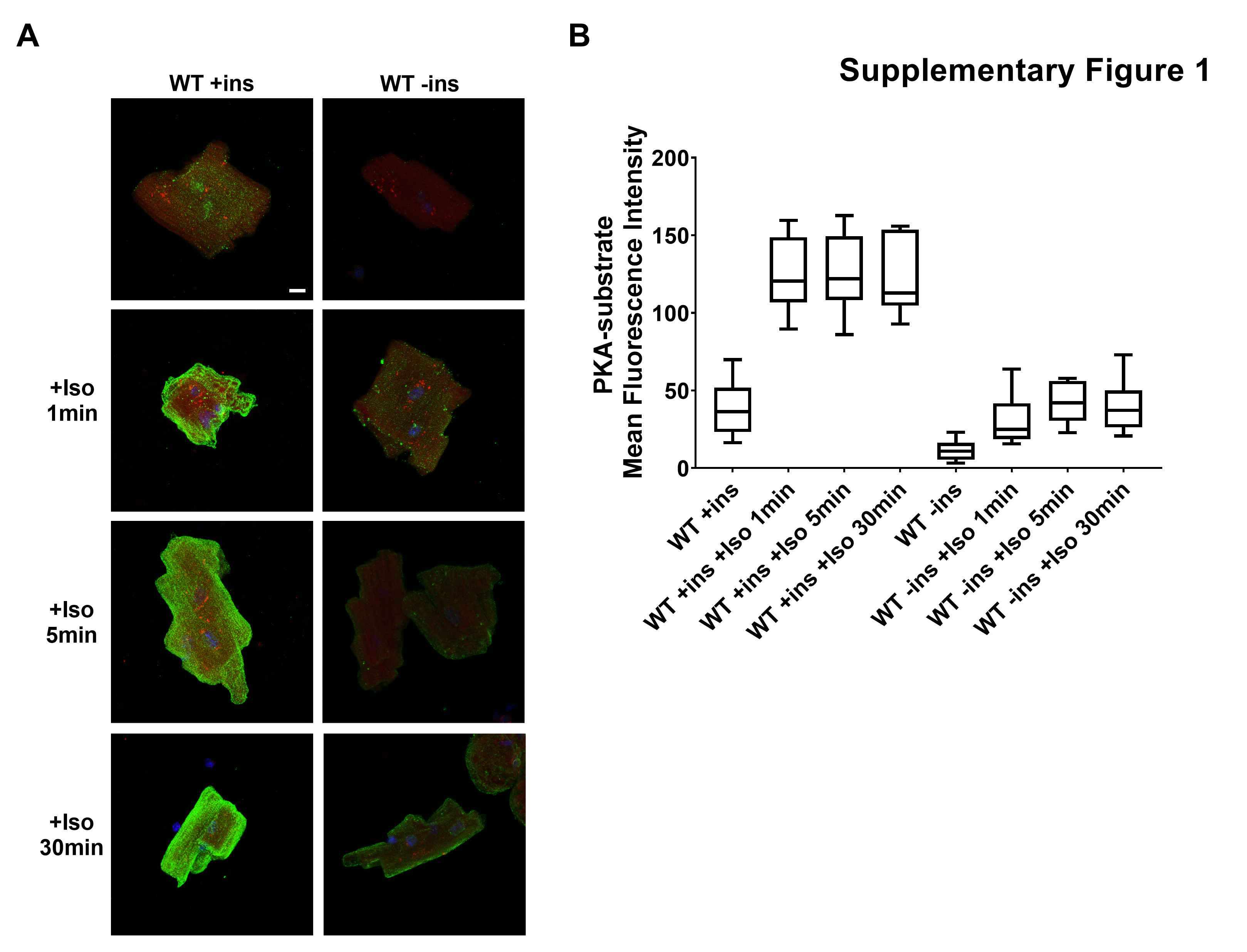

Supplement: S1 Fig — (A) ACMs from wild-type mice were incubated overnight in the presence or absence of insulin (ins) and treated with 0.25μM Isoproterenol for 1, 5, and 30min as indicated. Cells were fixed and stained with rabbit anti-PKA substrate antibody visualized with Alexa 488 anti-rabbit secondary and with Alexa 568 labeled phalloidin. Maximum intensity micrographs were acquired as described in Materials and Methods and a representative image for each condition is shown. Scale bar 10μm. (B) Quantitation of mean fluorescence intensity (MFI) for PKA-substrate are presented as whisker plots that encompass data from at least 18 cells, as detailed in Fig 1 (n = 3 biological replicates, and at least 6 cells per experiment). *, significant difference (p < .001) by one-way ANOVA with Tukey post hoc test. (TIF) [file pone.0231806.s001.tif]

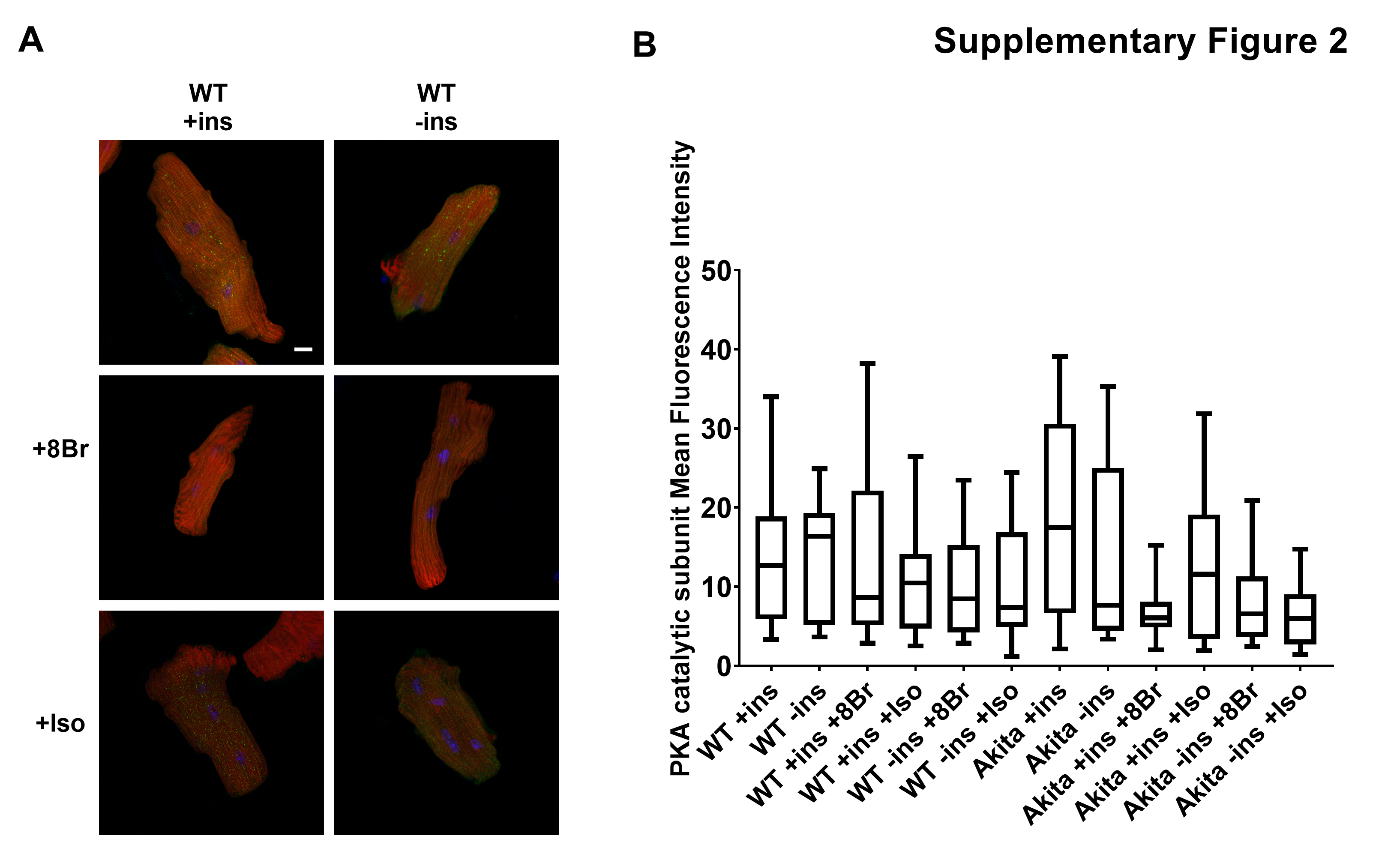

Supplement: S2 Fig — (A) ACMs from wild-type or Akita mice were incubated overnight in the presence or absence of insulin (ins) and treated with 0.25μM Isoproterenol or 250μM 8-bromo-cAMP for 30min as indicated. Cells were fixed and stained with rabbit anti-PKA catalytic subunit antibody visualized with Alexa 488 anti-rabbit secondary and with Alexa 568 labeled phalloidin. Maximum intensity micrographs were acquired as described in Materials and Methods and a representative image for each condition is shown. Scale bar 10μm. (B) Quantitation of mean fluorescence intensity (MFI) for PKA-substrate are presented as whisker plots that encompass data from at least 18 cells, as detailed in Fig 1 (n = 3 biological replicates, and at least 6 cells per experiment). (TIF) [file pone.0231806.s002.tif]

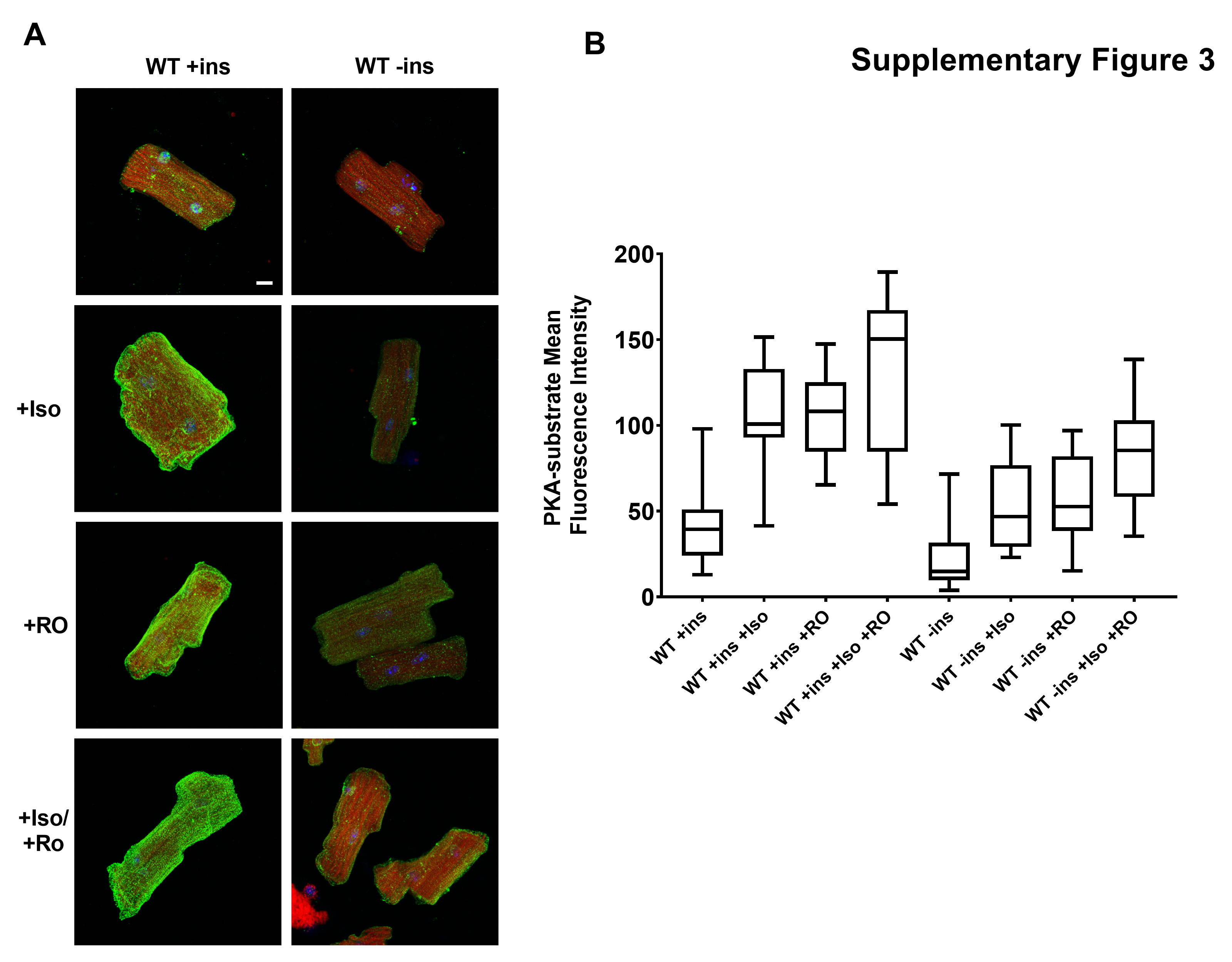

Supplement: S3 Fig — (A) ACMs from wild-type mice were incubated overnight in the presence or absence of insulin (ins) and treated with 0.25μM Isoproterenol and/or 10μM RO. Cells were fixed and stained with rabbit anti-PKA substrate antibody visualized with Alexa 488 anti-rabbit secondary and with Alexa 568 labeled phalloidin. Maximum intensity micrographs were acquired as described in Materials and Methods and a representative image for each condition is shown. Scale bar 10μm. (B) Quantitation of mean fluorescence intensity (MFI) for PKA-substrate are presented as whisker plots that encompass data from at least 18 cells, as detailed in Fig 1 (n = 3 biological replicates, and at least 6 cells per experiment). *, significant difference (p < .001) by one-way ANOVA with multiple comparisons using Tukey’s test. (TIF) [file pone.0231806.s003.tif]

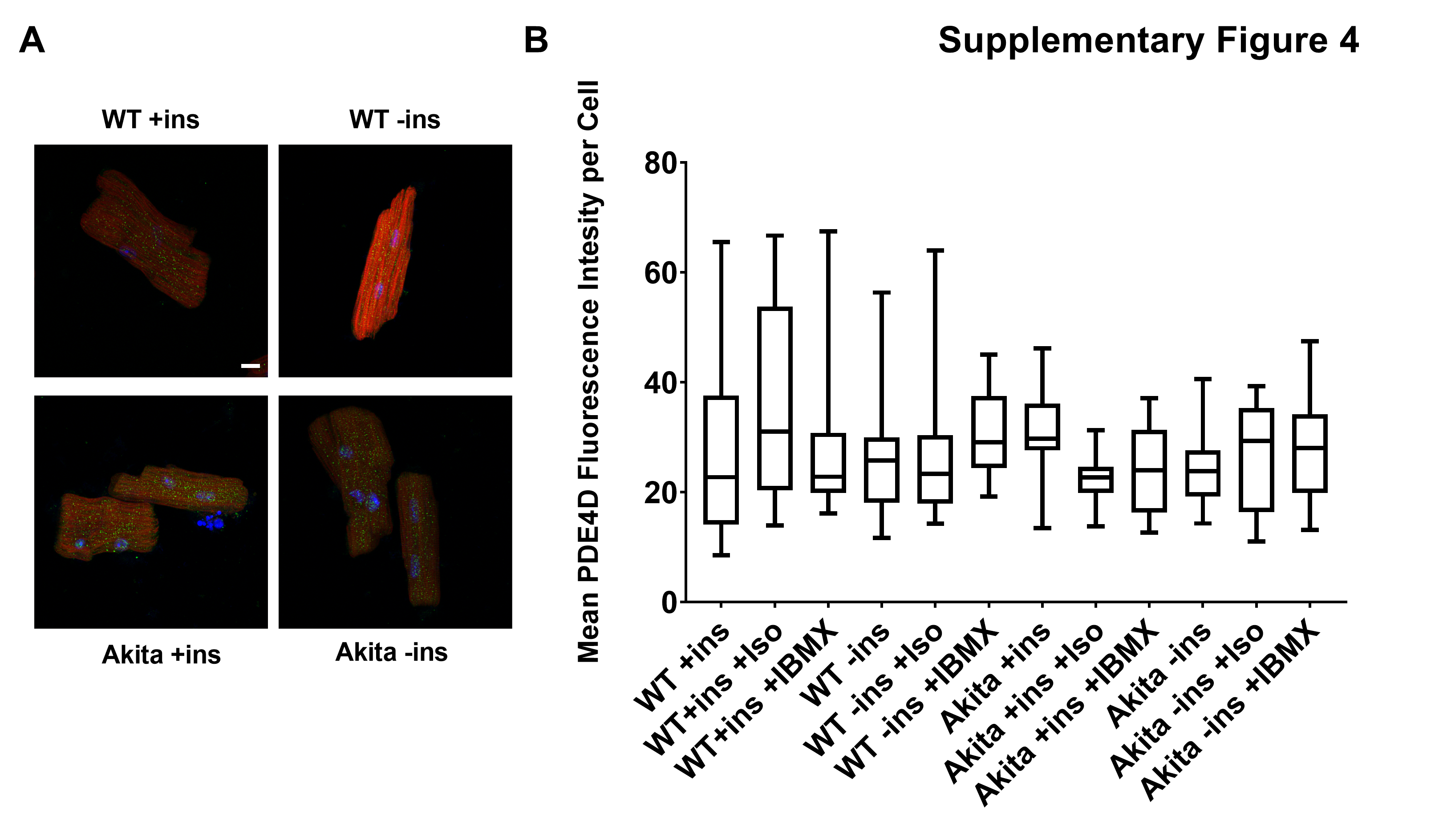

Supplement: S4 Fig — (A) Primary mouse cardiomyocytes from wild-type (C57-B6) or Akita were incubated overnight in the presence or absence of insulin (ins) and treated with 0.25μM Isoproterenol or 500μM IBMX for 30min. Cells were fixed and stained with rabbit anti-PDE4D antibody visualized with Alexa 488 anti-rabbit secondary and with Alexa 568 labeled phalloidin. Maximum intensity micrographs were acquired as described in Materials and Methods and a representative image for each condition is shown. Scale bar 10μm. (B) Quantitation of mean fluorescence intensity (MFI) for PDE4D are presented as whisker plots that encompass data from at least 18 cells, as detailed in Fig 1 (n = 3 biological replicates, and at least 6 cells per experiment). *, significant difference (p < .001) by one-way ANOVA with multiple comparisons using Tukey’s test. (TIF) [file pone.0231806.s004.tif]
