# Supplementary material for: Diabetes induced decreases in PKA signaling in cardiomyocytes: The role of insulin
Source: PLoS One. 2020 Aug 20;15(8):e0231806. doi: 10.1371/journal.pone.0231806 (PMC7444578; doi:10.1371/journal.pone.0231806)
Supplement: S1 Raw images — (PDF) [file pone.0231806.s005.pdf]

Raw Western Data

Figure 1C

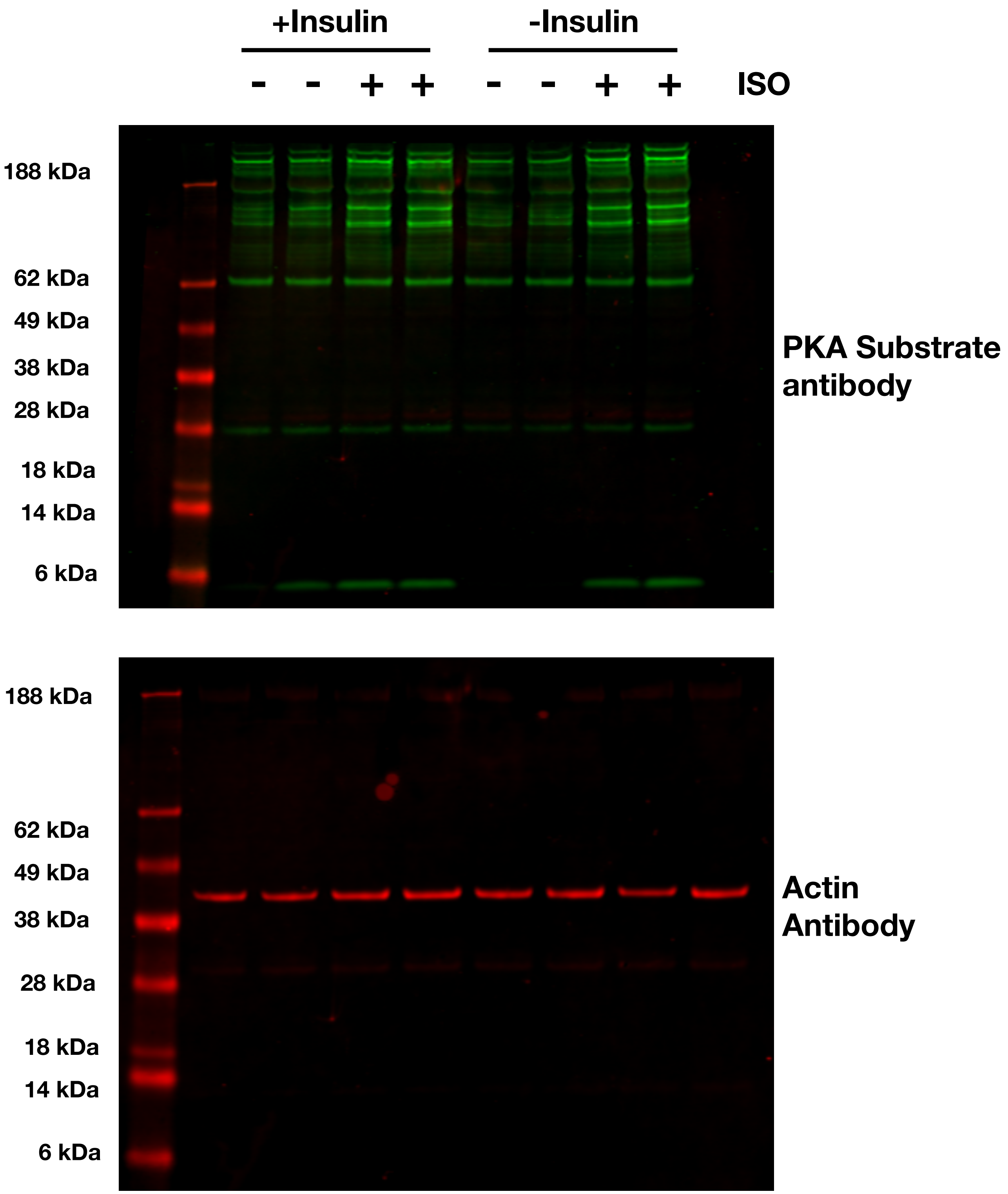

Raw Western blot data for **Fig 1C**. The *Top* shows PKA substrate antibody staining. The blot was imaged with a LI-COR Odyssey CLx instrument. Lane 1 (red bands) contains SeeBlue protein ladder (ThermoFisher). *Bottom: t*he blot was re-probed with an Actin antibody. For Figure 1C the blots were converted into a black and white image with a light background for easier visualization. No other enhancements or adjustments were made. The actin blot was cropped and converted to a black and white image with a light background for presentation.

Figure 7C

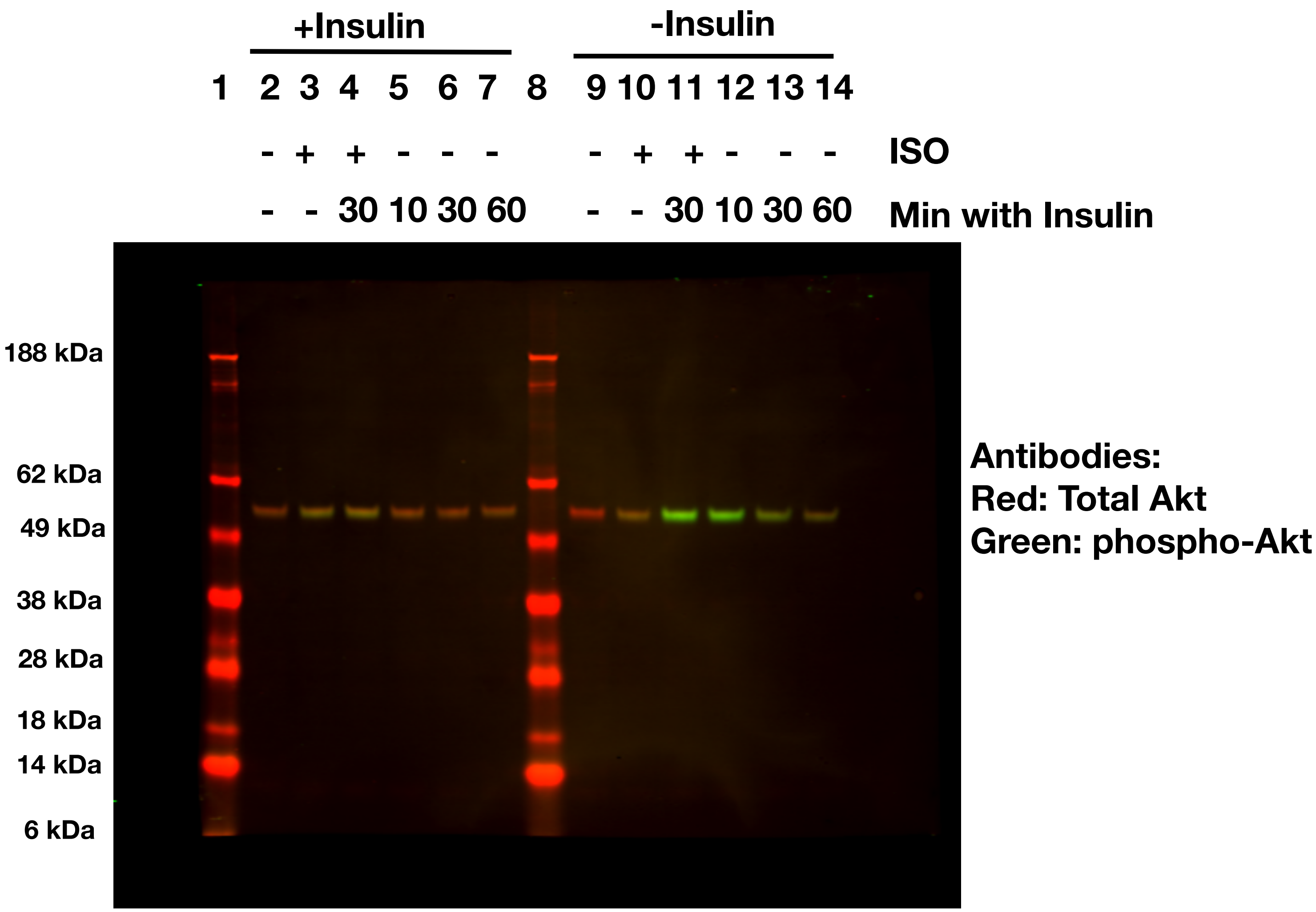

Raw Western blot data for **Fig 7C**. Adult mouse cardiomyocytes were cultured overnight with insulin (lanes 2-7) or without (lanes 9-14). ACMs were then treated with ISO and/or insulin as indicated. The blot was simultaneously probed with antibodies against phospho-Akt-S473 (green) and total Akt (red) and imaged with a LI-COR Odyssey CLx instrument. Lanes 1 and 8 (red bands) contain SeeBlue protein ladder (ThermoFisher). The image in Fig. 7C was cropped for presentation but otherwise unedited.
