# Supplementary material for: Diabetes induced decreases in PKA signaling in cardiomyocytes: The role of insulin
Source: PLoS One. 2020 Aug 20;15(8):e0231806. doi: 10.1371/journal.pone.0231806 (PMC7444578; doi:10.1371/journal.pone.0231806)
Supplement: Attachment — Submitted filename: Reviewer response.docx [file pone.0231806.s007.docx]

We’d like to thank the reviewers for their evaluation and constructive critiques of our original and revised manuscripts.

**Reviewer 2:** At this stage, I have one minor concern, it is still not clear what exactly is N in this study. Are the statistics are performed on biological repeats (n=3) or cells (n= approx. 30) as is shown on the example data point plot provided? If it is biological repeats were the cell data averaged for each animal? On the contrary, if the stats were performed on cells, the authors need to be wary of pseudoreplication. It is recommended that a line is added to the methods section to clarify this.

I apologize that this wasn’t clear. The experiments under a given condition were done on 3 cell preparations from 3 hearts. The statistics were performed on the sum of all the cells that were imaged. I have added additional information in the Methods section and in each of the Figure Legends to make this clear. I agree that n= appox. 30 can be considered as pseudoreplication, or as we considered them repeated measurements, and this could be a problem. However, when the cells from a given preparation are averaged and analyzed as n=3 similar conclusions are still reached. This information has been added in the “Statistical analysis” section in the Methods. For example, here is Figure 1 plotted with all the cell data shown or with the data plotted as averages of the 3 cell preparations. We believe that showing the whisker plots encompassing all the cells more clearly demonstrates the data distribution and variability intrinsic to the method.
